# CD38-Specific Biparatopic Heavy Chain Antibodies Display Potent Complement-Dependent Cytotoxicity Against Multiple Myeloma Cells

**DOI:** 10.3389/fimmu.2018.02553

**Published:** 2018-11-19

**Authors:** Kerstin Schütze, Katharina Petry, Julia Hambach, Niklas Schuster, William Fumey, Levin Schriewer, Jana Röckendorf, Stephan Menzel, Birte Albrecht, Friedrich Haag, Catelijne Stortelers, Peter Bannas, Friedrich Koch-Nolte

**Affiliations:** ^1^Institute of Immunology, University Medical Center Hamburg-Eppendorf, Hamburg, Germany; ^2^Department of Radiology, University Medical Center Hamburg-Eppendorf, Hamburg, Germany; ^3^Ablynx NV, Ghent, Belgium

**Keywords:** complement-dependent cytotoxicity, CD38, multiple myeloma, nanobody, heavy chain antibody, antibody engineering, biparatopic antibodies

## Abstract

CD38 is overexpressed by multiple myeloma cells and has emerged as a target for therapeutic antibodies. Nanobodies are soluble single domain antibody fragments derived from the VHH variable domain of heavy chain antibodies naturally occurring in camelids. We previously identified distinct llama nanobodies that recognize three non-overlapping epitopes of the extracellular domain of CD38. Here, we fused these VHH domains to the hinge, CH2, and CH3 domains of human IgG1, yielding highly soluble chimeric llama/human heavy chain antibodies (hcAbs). We analyzed the capacity of these hcAbs to mediate complement-dependent cytotoxicity (CDC) to CD38-expressing human multiple myeloma and Burkitt lymphoma cell lines. Combinations of two hcAbs that recognize distinct, non-overlapping epitopes of CD38 mediated potent CDC, in contrast to the hcAb monotherapy with only weak CDC capacity. Similarly, combining daratumumab with a hcAb that recognizes a non-overlapping epitope resulted in dramatically enhanced CDC. Further, introducing the E345R HexaBody mutation into the CH3 domain strongly enhanced the CDC potency of hcAbs to CD38-expressing cells. Exploiting their high solubility, we genetically fused two distinct nanobodies into heteromeric dimers via a flexible peptide linker and then fused these nanobody dimers to the hinge, CH2 and CH3 domains of human IgG1, yielding highly soluble, biparatopic hcAbs. These biparatopic hcAbs elicited CDC toward CD38-expressing myeloma cells more effectively than daratumumab. Our results underscore the advantage of nanobodies vs. pairs of VH and VL domains for constructing bispecific antibodies. Moreover, the CD38-specific biparatopic heavy chain antibodies described here represent potential new powerful therapeutics for treatment of multiple myeloma.

## Introduction

CD38 is overexpressed by multiple myeloma and other hematological tumors and has attracted interest as a target for therapeutic antibodies (1–4). CD38 is a cell surface ecto-enzyme that metabolizes NAD^+^ released from damaged cells in inflammation (5). In concert with CD203 and CD73, CD38 contributes to the conversion of NAD^+^ to immunosuppressive adenosine in the tumor microenvironment (6, 7). By suppressing effector T cell responses, CD38 may thereby promote tumor growth (5, 8). The conventional CD38-specific monoclonal antibody daratumumab was generated from CD38-immunized transgenic mice that carry genomic loci encoding human IgH and IgL (9). Daratumumab has proven high therapeutic efficacy in multiple myeloma (3, 10).

Complement-dependent cytotoxicity (CDC) is an important mechanism for the killing of tumor cells (11–13). CDC is initiated when complement factor 1 (C1q) binds to antibodies on the cell surface. It has been recognized that monospecific IgG antibodies are generally ineffective at inducing CDC, while IgM and combinations of non-crossreactive IgG molecules induce potent CDC (14–17). Modeling and mutagenesis studies suggest that IgG hexamer formation facilitates efficient binding and activation of C1q (18). Amino acid substitutions in the CH3 domain of daratumumab that enhanced the formation of IgG hexamers were found to enhance the binding of C1q to CD38 on the cell surface. These so called HexaBody mutations also enhanced CDC by daratumumab (18, 19).

The variable domain of heavy chain antibodies that naturally occur in camelids is called VHH or nanobody (Nanobody® is a trademark of Ablynx). Nanobodies exhibit several advantages over conventional antibodies (20–24). The single domain format of nanobodies greatly facilitates the construction of bispecific and biparatopic dimers by genetically linking two nanobodies with a flexible peptide linker (25–30). Genetic fusion of a nanobody to the hinge, CH2 and CH3 domains of human IgG1 yields highly soluble llama/human chimeric heavy chain antibodies (hcAbs) (31–33). At half the size of a conventional antibody (75 vs. 150 kDa), hcAbs may penetrate tissues better than conventional antibodies (32). To date, more than 2,000 patients and healthy subjects have received nanobodies in clinical studies without any adverse side effects (34–36). The European Commission recently granted marketing authorization for the first nanobody-based drug, Caplacizumab (Cablivi™), a nanobody-dimer directed against von Willebrand factor, for the treatment of acquired thrombotic thrombocytopenic purpura (aTTP), a rare blood clotting disorder (37).

The goal of this study was to assess the capacity of CD38-specific hcAbs to induce CDC to CD38-expressing multiple myeloma cells. Our results show that the combination of two CD38 hcAbs elicits potent CDC, provided the two hcAbs recognize distinct epitopes. We sought to exploit the high solubility of nanobodies to construct highly soluble biparatopic nanobody-based hcAbs that contain a tandem pair of CD38-specific nanobodies recognizing non-overlapping epitopes. Remarkably, these biparatopic hcAbs show higher CDC potency than daratumumab and therefore hold promise as novel therapeutics for the treatment of multiple myeloma.

## Results

### Individual CD38-specific hcAbs induce little if any CDC

**Figure 1:**
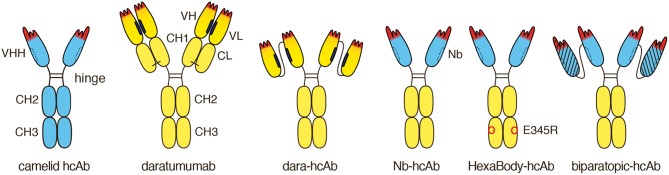
Schematic diagram of heavy chain antibodies (hcAbs) used in this study. Naturally occurring camelid hcAbs lack the CH1 domain and light chains. The antigen binding module of these hcAbs is composed of a single highly soluble variable domain (VHH) that is linked directly to the hinge. Like other conventional antibodies, daratumumab is composed of two IgG1 heavy chains and two kappa light chains. The antigen binding module of daratumumab is composed of two non-covalently associated variable domains, VH, and VL. The proper orientation of these domains is mediated by a hydrophobic interface (indicated in black) and is further stabilized by the disulfide linked CL and CH1 domains. We genetically fused the VH and VL domains of daratumumab via a flexible peptide linker and further fused this single chain variable fragment (scFv) to the hinge, CH2, and CH3 domains of IgG1, generating dara-hcAb, corresponding to the format of camelid hcAbs. The proper orientation of the antigen recognition module in this construct is mediated solely by the hydrophobic interface between the two V domains. We fused distinct CD38-specific VHH domains to the hinge, CH2 and CH3 domains of human IgG1, generating Nb-hcAbs, i.e., chimeric llama/human IgG1 hcAbs. A recombinant VHH domain or nanobody (Nb) is highly soluble and does not show any tendency to associate with light chains or any other hydrophobic proteins (the hydrophilic face corresponding to the hydrophobic VL-interface of conventional antibodies is indicated by a dashed line). We introduced the E345R HexaBody mutation into some hcAbs, generating HexaBody-hcAbs. We further exploited the inherent solubility of VHHs to generate biparatopic hcAbs by fusing two distinct CD38-specific VHHs via a flexible G4Sn linker and further fusing such dimers to the hinge, CH2 and CH3 domains of human IgG1.

**Figure 2:**
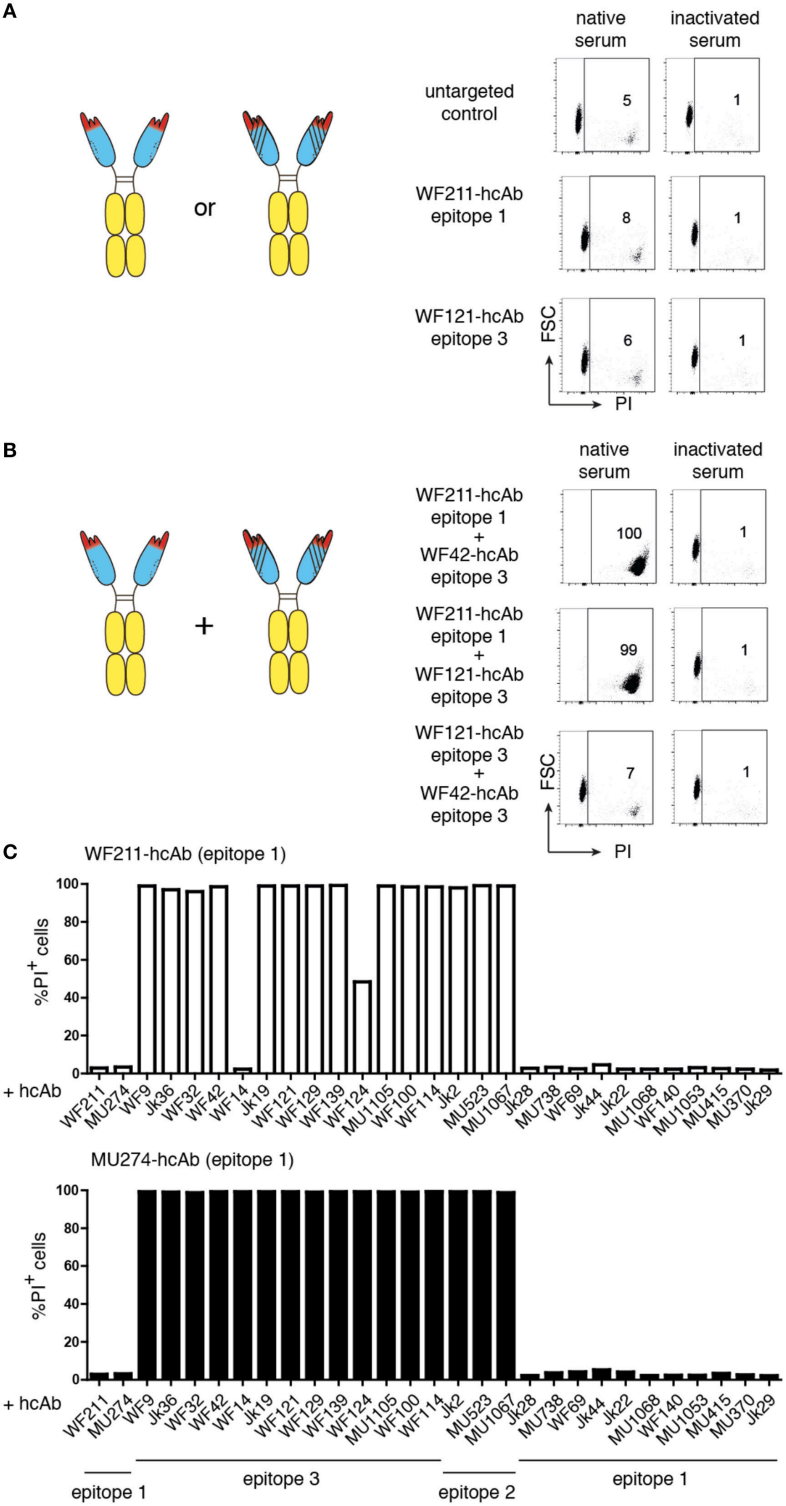
Combinations of two hcAbs recognizing non-overlapping epitopes of CD38 are potent inducers of CDC. CA-46 cells were incubated for 60 min at 37°C in the presence of of saturating amounts (10–30 nM) of individual hcAbs **(A)** or with combinations of two hcAbs **(B)** and native serum or inactivated serum (preincubated for 30 min at 56°C to inactivate complement components). Cells were washed and resuspended in PBS containing BSA and propidium iodide (PI) before analysis by flow cytometry. **(A,B)** The schematics illustrate the CD38-specific hcAbs used in this experiment. Representative FACS plots illustrate the gating strategy used to determine the percentages of dead cells (PI +, FSC = forward scatter low). **(C)** Bar diagrams showing % of PI-positive cells of samples treated with a combination of either WF211-hcAb or MU274-hcAb and the hcAbs indicated below. Results are representative of three similar experiments.

In order to generate CD38-specific heavy chain antibodies, we genetically fused the nanobody coding sequence to the coding sequence for the hinge, CH2, and CH3 domains of human IgG1 (Figure 1). We tested the capacity of individual CD38-specific hcAbs to induce CDC to CD38-expressing LP-1 or CA-46 tumor cells in the presence of human serum as a source of complement (Figure 2). After 1 h incubation at 37°C cells were analyzed by flow cytometry for uptake of the DNA-staining dye propidium iodide as a marker for cell death. The results reveal that individual hcAbs show little if any capacity to induce CDC (Figure 2A).

### Combinations of two hcAbs recognizing non-overlapping epitopes of CD38 are potent inducers of CDC

It has been shown that combinations of non-crossreactive IgG antibodies can induce potent CDC (15–17). We therefore tested whether combinations of two distinct CD38-specific hcAbs could induce CDC. Indeed, certain combinations of hcAbs induced potent CDC, whereas other hcAb combinations were as ineffective as individual hcAbs (Figures 2B,C). Pre-incubating the serum for 30 min at 56°C abrogated cytotoxicity, indicating that killing was dependent on active complement components.

We had previously assigned the 22 nanobodies to one of three distinct epitopes of CD38 on the basis of cross-blockade and sequential binning analyses (38). Taking these epitope assignments into consideration, a clear pattern emerges: Any combination of two hcAbs that recognize non-overlapping epitopes elicits very potent CDC whereas any combination of two hcAbs that recognize overlapping epitopes elicits little if any CDC (Table 1).

**Table 1:** Combinations of two CD38-specific hcAbs recognizing distinct epitopes induce potent CDC.

**Epitope**	**–**	**JK2-hcAb**	**MU1067-hcAb**	**WF211-hcAb**	**MU274-hcAb**	**JK36-hcAb**	**WF100-hcAb**
2	JK2-hcAb	5	4	99	100	100	96
2	MU1067-hcAb	4	5	100	99	100	96
2	MU523-hcAb	4	5	100	100	100	96
1	MU738-hcAb	100	100	2	3	100	97
1	JK44-hcAb	100	100	3	4	100	98
1	JK29-hcAb	99	100	1	2	100	97
1	MU1068-hcAb	100	100	1	1	100	96
1	MU415-hcAb	100	100	2	3	100	96
1	JK22-hcAb	99	100	2	4	100	90
1	WF211-hcAb	100	100	3	3	100	37
1	MU1053-hcAb	100	100	2	1	100	54
1	MU370-hcAb	100	100	1	2	100	97
1	MU274-hcAb	100	100	2	2	100	95
1	JK28-hcAb	100	100	2	1	100	53
3	WF124-hcAb	96	100	50	100	4	2
3	WF121-hcAb	99	99	99	100	4	2
3	WF42-hcAb	95	98	99	100	8	2
3	JK19-hcAb	100	100	100	100	5	2
3	JK36-hcAb	100	100	98	100	4	2
3	WF100-hcAb	100	100	99	100	5	2
3	WF9-hcAb	100	100	100	100	4	2
3	WF14-hcAb	81	97	2	100	4	2

*Numbers indicate the percentage of PI positive cells, 60 min after incubation of CA-46 cells in the presence of saturating amounts (10–30 nM) of the indicated combinations of hcAbs and human serum. Numbering of binding epitopes is according to Fumey et al. (38)*.

### Combination of daratumumab with a CD38-specific hcAb that binds a distinct epitope of CD38 enhances it's CDC potency

**Figure 3:**
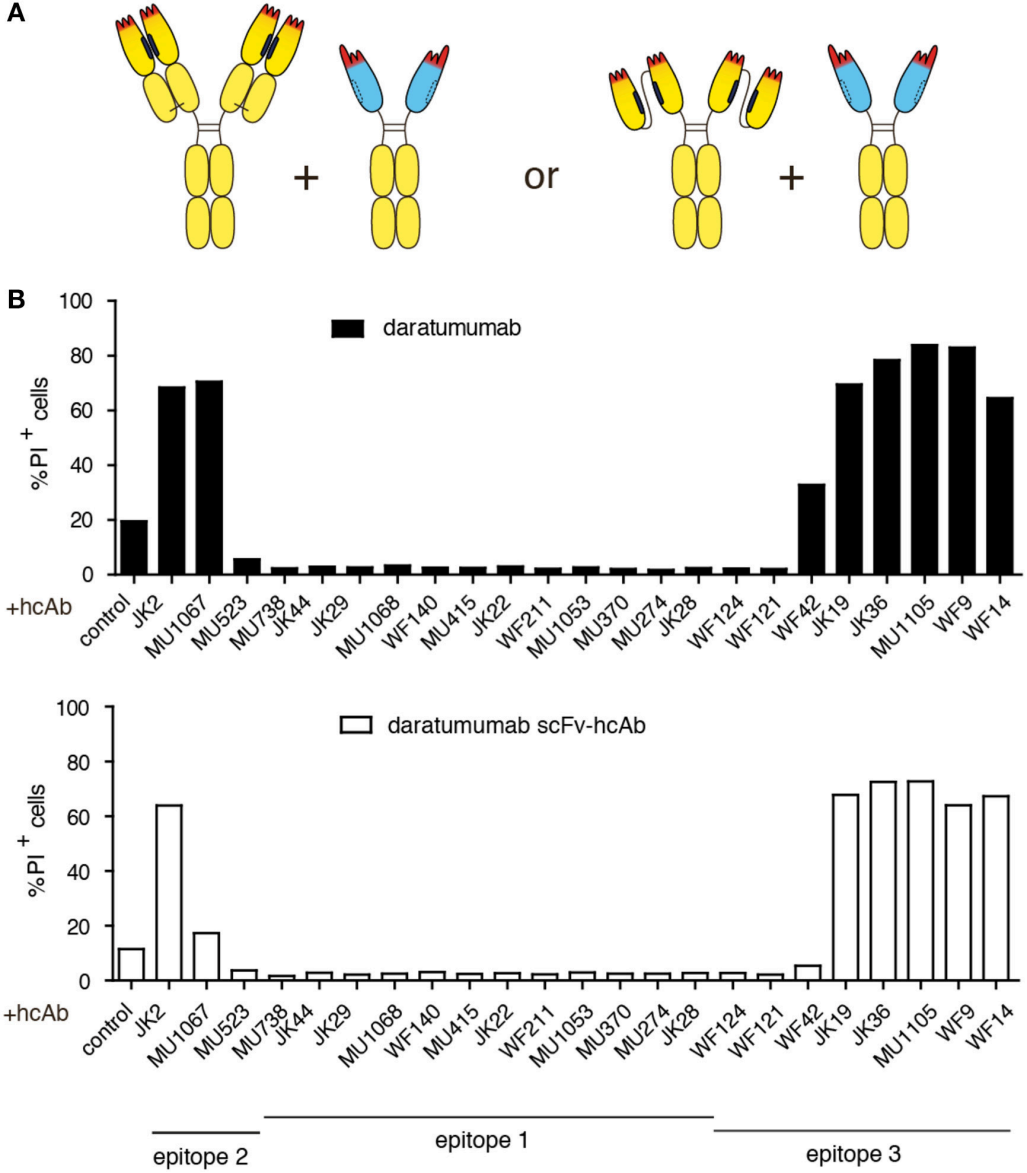
Combination of daratumumab with an anti-CD38 hcAb that binds a distinct epitope on CD38 enhances its CDC potency. CA-46 cells were incubated for 60 min at 37°C in the presence of Ab combinations (50 nM each) containing either daratumumab or a scFv-hcAb form of daratumumab and a CD38-specific hcAb and 15% (v/v) of native human serum. Cells were washed and resuspended in PBS containing BSA and propidium iodide before analysis by flow cytometry. **(A)** Schematic illustrating the constructs used in this experiment. **(B)** Bar diagrams showing % of PI-positive cells ± standard deviation of three samples treated in parallel with the indicated hcAbs. Results are representative of four similar experiments.

We next tested whether any of our CD38-specific hcAbs could elicit potent CDC also in combination with the benchmark therapeutic antibody daratumumab (Figure 3). The results show that only certain hcAbs complement daratumumab to induce potent CDC. Considering our previous assignment of nanobody epitopes relative to that of daratumumab (38), a similar clear pattern again emerges: CD38-specific hcAbs carrying a nanobody that binds independently of daratumumab elicit potent CDC when combined with daratumumab. In contrast, CD38-specific hcAbs carrying a nanobody that binds an epitope overlapping with daratumumab elicit little if any CDC when combined with daratumumab (Table 2).

**Table 2:** Combinations of daratumumab with a hcAb recognizing a distinct epitope induce potent CDC.

**Epitope**	**hcAb**	**Daratumumab**
2	JK2-hcAb	86
2	MU1067-hcAb	68
2	MU523-hcAb	60
1	MU738-hcAb	9
1	JK44-hcAb	17
1	JK29-hcAb	11
1	MU1068-hcAb	6
1	MU415-hcAb	8
1	JK22-hcAb	5
1	WF211-hcAb	4
1	MU1053-hcAb	5
1	MU370-hcAb	7
1	MU274-hcAb	6
1	JK28-hcAb	6
3	WF124-hcAb	29
3	WF121-hcAb	30
3	WF42-hcAb	80
3	JK19-hcAb	95
3	JK36-hcAb	65
3	WF100-hcAb	93
3	WF9-hcAb	94
3	WF14-hcAb	65

*Numbers indicate the percentage of PI positive cells, 90 min after incubation of LP-1 cells in the presence of the indicated combinations of daratumumab with a CD38-specific hcAb and human serum. Numbering of binding epitopes is according to Fumey et al. (38)*.

In analogy to the nanobody-based heavy chain antibodies, we also constructed a hcAb version of daratumumab by fusing the VH and VL domains of daratumumab via a flexible Gly-Ser linker and further fusing this scFv to the hinge, CH2 and CH3 domains of human IgG1. In CDC assays, the same hcAbs that enhanced the CDC potency of daratumumab also enhanced the CDC potency of this dara-hcAb (Figure 3B).

### Introduction of the E345R HexaBody mutation enhances the CDC potency of CD38-specific hcAbs

**Figure 4:**
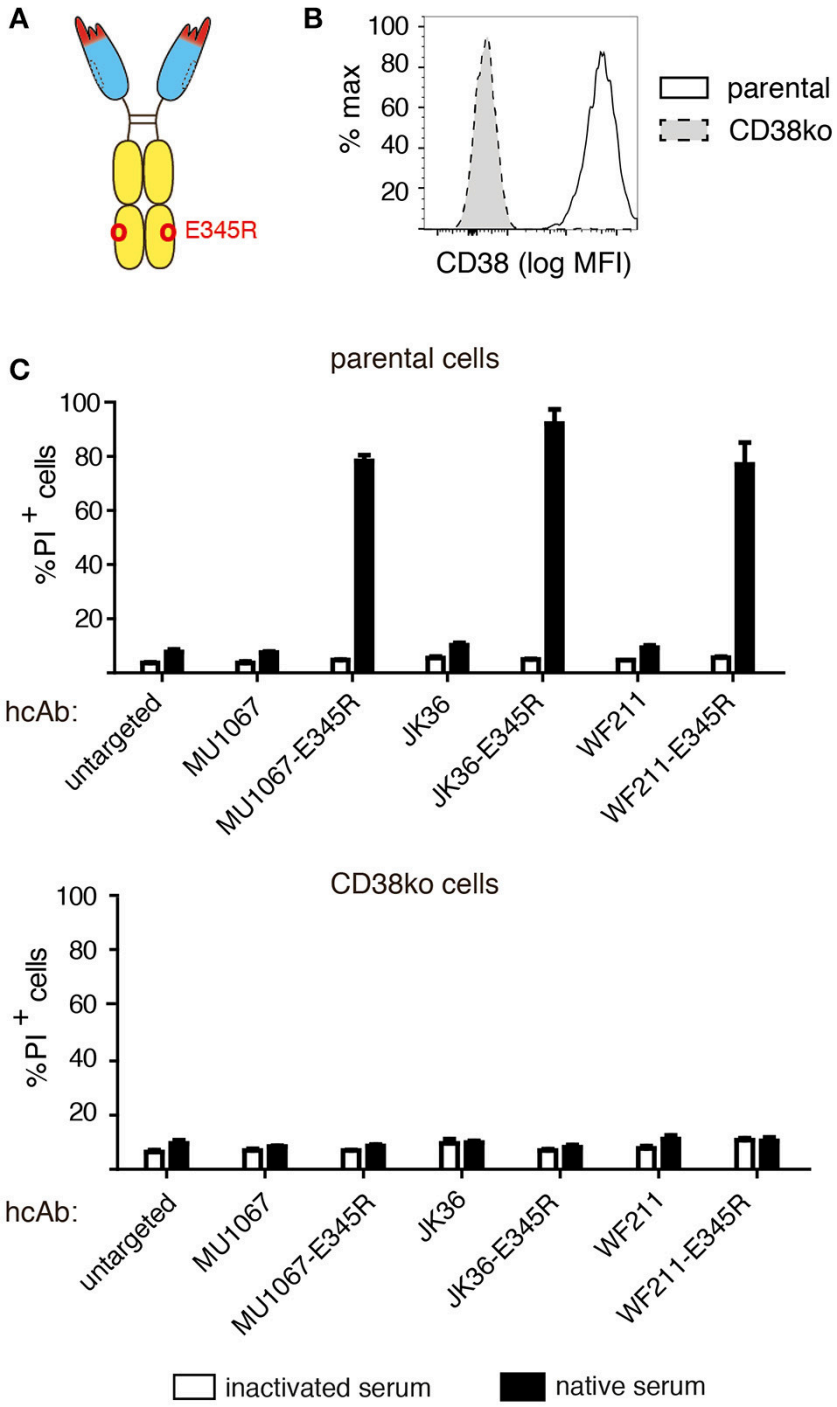
Introduction of the E345R HexaBody mutation enhances the CDC potency of CD38-specific hcAbs. **(A)** Schematic illustration of the hcAb E345R mutant that facilitates Fc-hexamerization used in this experiment. **(B)** The CD38 gene was stably inactivated in LP-1 cells using CRISPR/Cas9 technology. Cells were stained with AF647-conjugated Nb JK36 and analyzed by flow cytometry. **(C)** Parental and CD38ko LP-1 cells were incubated for 60 min at 37°C with the indicated CD38-specific hcAbs (100 nM) and 15% v/v native human serum. Cells were stained with propidium iodide and analyzed by flow cytometry. Bar diagrams showing % of PI-positive cells ± standard deviation of three samples treated in parallel with the indicated hcAbs. Results are representative of four similar experiments.

It has been shown that certain amino acid substitutions in the C1q binding face of daratumumab enhance the tendency of daratumumab to spontaneously form hexamers (18). These so called HexaBody mutations enhanced the CDC potency of daratumumab (19). We aimed to determine whether a HexaBody mutation would similarly enhance the CDC potency of CD38-specific hcAbs. We therefore introduced the E345R mutation into the CH3 domain of our hcAbs and analyzed the capacity of these HexaBody hcAbs to induce CDC. The results, indeed, reveal an enhanced CDC potency of the HexaBody hcAbs over their parental counterparts (Figure 4). LP-1 cells in which the CD38 gene had been inactivated by CRISPR/Cas9 technology were resistant to CDC by HexaBody hcAbs, indicating that binding to CD38 is essential for induction of CDC.

### Combining nanobodies directed to two distinct epitopes on CD38 in a biparatopic hcAb induces potent CDC

**Figure 5:**
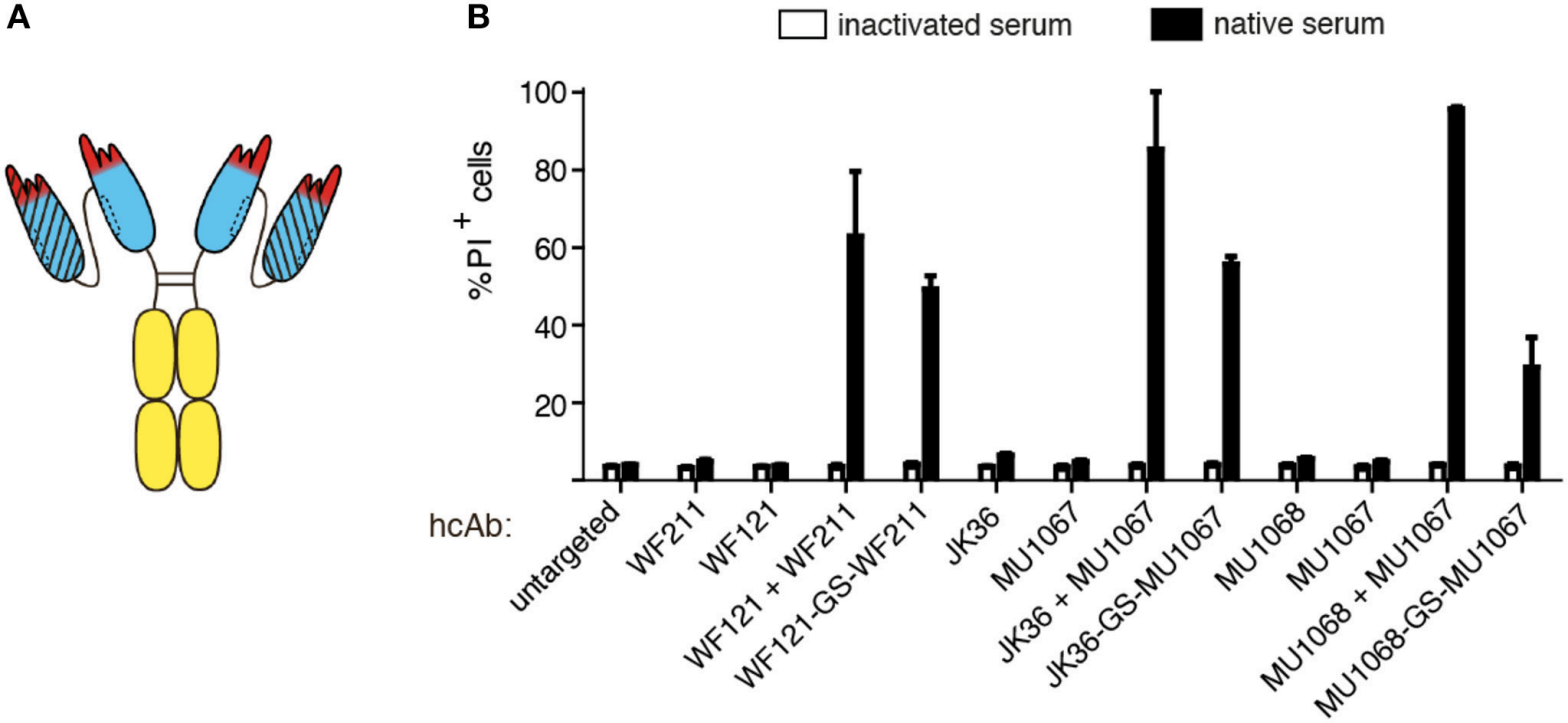
Combining two nanobodies directed to distinct epitopes of CD38 in a biparatopic hcAb induces potent CDC. LP-1 cells were incubated for 60 min at 37°C with an untargeted control hcAb or with the indicated monospecific or biparatopic hcAbs (100 nM) and either native human serum or inactivated serum. Cells were washed, stained with propidium iodide and analyzed by flow cytometry. **(A)** Schematic illustrating the biparatopic hcAbs used in this experiment. **(B)** Induction of CDC by biparatopic CD38-specific hcAbs combining two nanobodies with non-overlapping epitopes (“-GS-”) in comparison to the combinations of the respective hcAbs (“+”). Bar diagrams showing % of PI-positive cells ±standard deviation of three samples treated in parallel with the indicated hcAbs. Results are representative of four similar experiments.

The soluble nature of nanobodies allows easy reformatting of nanobodies into homo- and heteromeric dimers by linking the C-terminus of one nanobody to the N-terminus of another nanobody by a flexible peptide linker [e.g., (G4S)n]. Moreover, such nanobody dimers can be fused to the hinge, CH2, and CH3 domains of human IgG1 to generate tetravalent bispecific or biparatopic hcAbs (32). In order to determine whether the potent CDC induction capacity of certain hcAb combinations could be combined into a single molecule, we constructed biparatopic hcAbs containing two nanobodies that recognize distinct epitopes of CD38. These biparatopic hcAbs were produced at high yield as soluble proteins in transiently transfected HEK-6E cells. The results of CDC assays reveal that biparatopic hcAbs indeed induce potent CDC as single reagents (Figure 5).

### Biparatopic CD38-specific hcAbs have higher CDC potency than daratumumab

**Figure 6:**
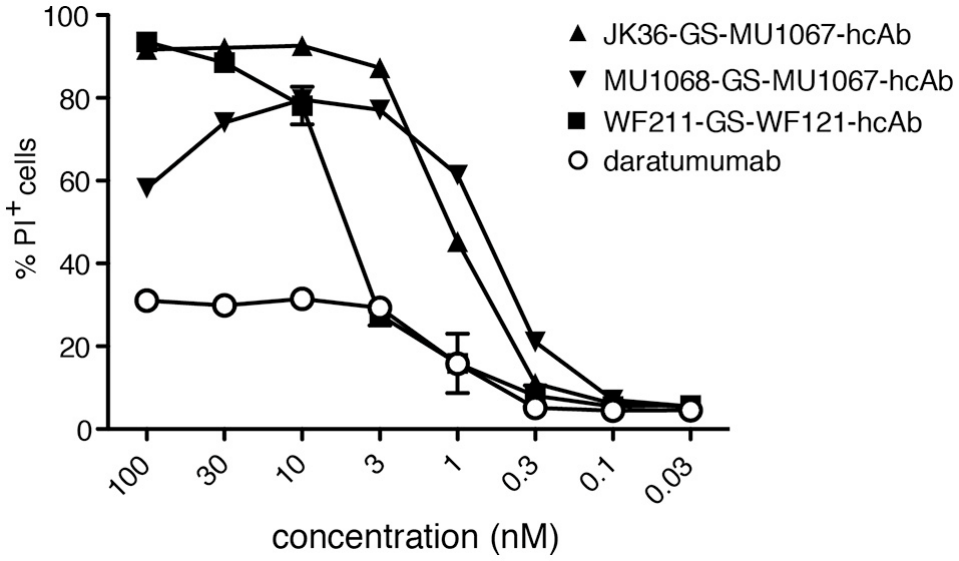
Biparatopic CD38-specific hcAbs combining nanobodies directed to two distinct epitopes on CD38 have higher CDC potency than daratumumab. CA-46 cells were incubated for 60 min at 37°C with titrated amounts of the indicated hcAbs or daratumumab and native human serum. Cells were washed, stained with propidium iodide and analyzed by flow cytometry. Results are representative of three similar experiments. Data are averages ±SD (*n* = 3).

In order to further compare the CDC potencies of daratumumab and our biparatopic hcAbs, we performed CDC-assays with titrated amounts of antibodies (Figure 6). The results show that the biparatopic hcAbs are much more potent than daratumumab at inducing CDC.

## Discussion

**Figure 7:**
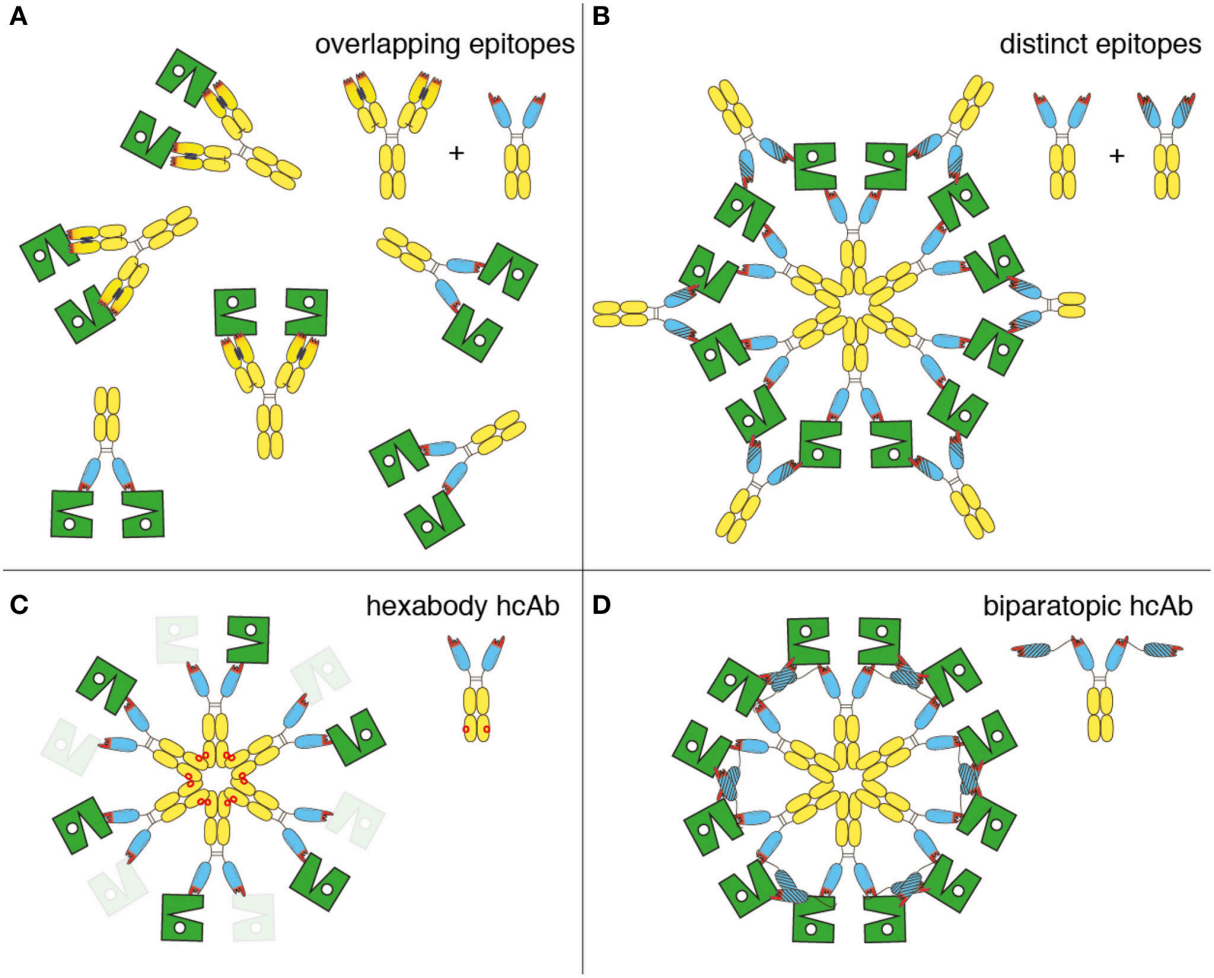
Schematic diagram illustrating the proposed molecular mechanism for the enhanced CDC by antibody combinations, HexaBody hcAbs, and biparatopic hcAbs. The extracellular enzymatic domain of CD38 is illustrated schematically in green, daratumumab and nanobody-based hcAbs are represented by the symbols used in other figures. **(A)** Single antibodies or combinations of antibodies (conventional or heavy chain) that recognize an overlapping epitope of CD38 can maximally crosslink two CD38 molecules. These CD38 dimers are not assembled into higher order oligomers and therefore show little if any capacity to bind C1q and to induce CDC. **(B)** Combinations of antibodies recognizing distinct epitopes of CD38 (conventional or heavy chain) facilitate formation of oligomers. **(C)** Introduction of the E345R hexabody mutation facilitates formation of hexameric antibody clusters on the cell surface. It remains to be determined whether HexaBody hcAbs are effective when binding monovalently (indicated by masked CD38), as demonstrated for daratumumab (18). **(D)** Biparatopic heavy chain antibodies containing nanobodies that recognize distinct epitopes of CD38 facilitate the formation of oligomers, since the two genetically fused VHH domains can bind to two different molecules of CD38.

Our results confirm the finding that combinations of two distinct, non-crossreactive IgG antibodies induce CDC more potently than monospecific IgG (15–17) and provide further insight into the molecular mechanism of this phenomenon. The schematic diagrams shown in Figure 7 present hypothetical models that need to be tested in more detail: Binding of a monospecific hcAb, moAb, or a combination of Abs that recognize an overlapping epitope of CD38 can maximally crosslink two CD38 molecules on the cell surface (Figure 7A). Addition of a second hcAb that binds to an epitope distinct from that of the first hcAb can crosslink two or more CD38 dimers connected by the first hcAb, thereby facilitating the formation of C1q-activating oligomers (Figure 7B). The E345R HexaBody mutation (18) enhances the CDC potency of hcAbs by facilitating formation of hexamers on the cell surface (Figure 7C). It is not known whether HexaBody hcAbs are also effective when binding monovalently as has been demonstrated for daratumumab (18). Remarkably, fusing two CD38-specific nanobodies that recognize distinct epitopes of CD38 into a biparatopic hcAb also results in potent CDC, likely reflecting the capacity of such biparatopic hcAbs to efficiently induce the formation of clusters (Figure 7D).

**Figure 8:**
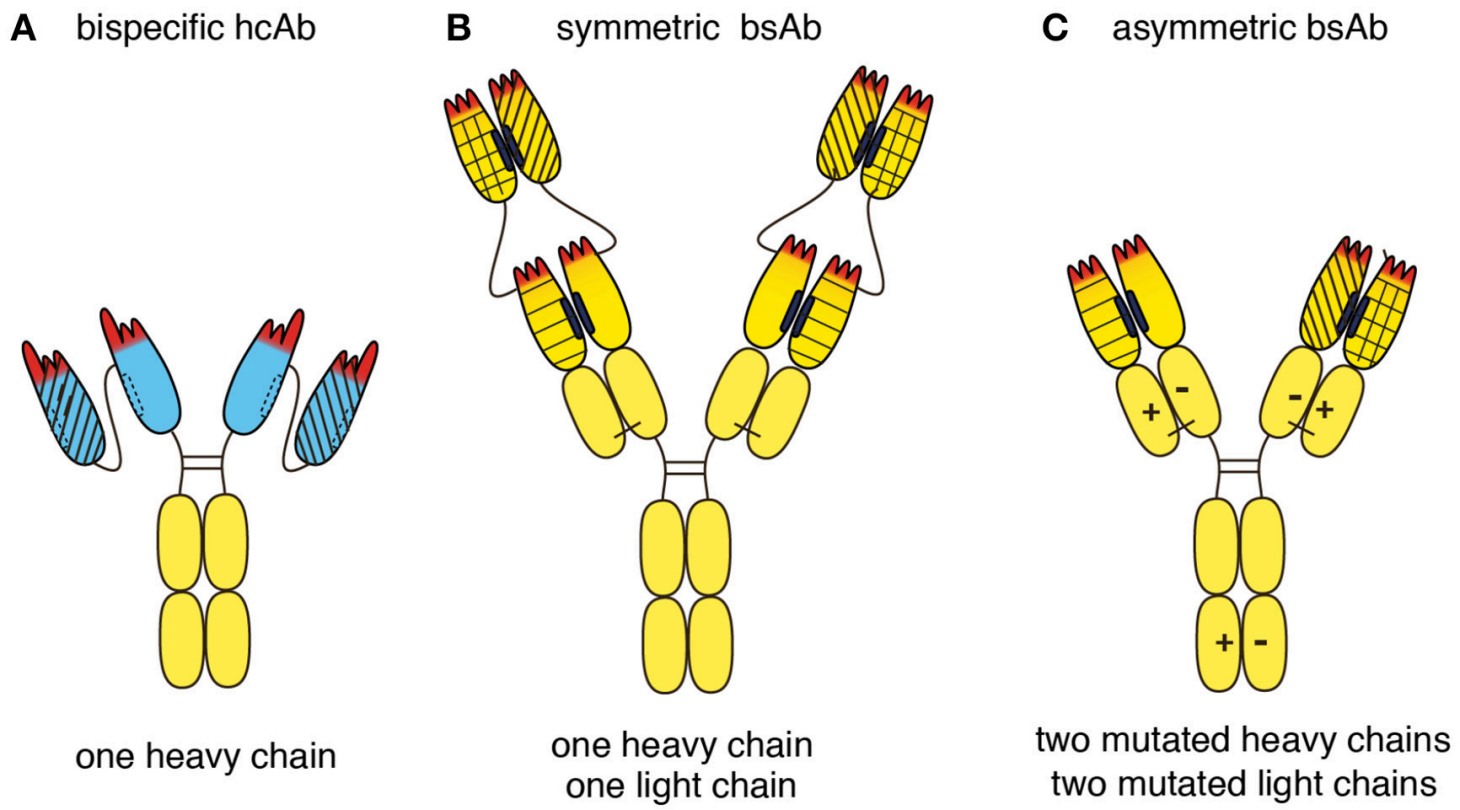
Schematic diagram illustrating the structural advantages of a biparatopic hcAb over a bispecific conventional moAb. **(A)** Llama VHH domains are depicted in blue, human Ig domains are depicted in yellow. Biparatopic nanobody-based hcAbs are composed of two identical heavy chains, each carrying two soluble VHH domains connected by a peptide linker. Biparatopic hcAbs therefore do not have any chain-pairing problem. **(B)** Symmetric bsAbs such as dual-variable-domain bsAbs are composed of two identical heavy chains and two identical light chains. Each of these chains is N-terminally extended by an additional V domain. The structural advantage of a biparatopic heavy chain antibody over such a dual-variable-domain bsAb lies in the inherent high stability and solubility of each VHH vs. the greater instability of each VH-VL pair. **(C)** Asymmetric bsAbs with the regular IgG architecture typically are composed of two distinct heavy chains and two distinct light chains. Mutations need to be introduced into both heavy chains to avoid unwanted homomeric pairing of heavy chains. Such mutations facilitate heteromeric pairing of heavy chains, e.g., by electrostatic pairing as indicated here (“+” and “–”), knob in hole, or CH3-repulsion. Similarly, unwanted pairing of light chains to the 'wrong' heavy chain can be minimized by introducing mutations into both light chains, e.g., electrostatic pairing as indicated here. Other strategies to minimize mispairing of light and heavy chains include swapping of CH1 and CL domains in one of the antibodies, using a fixed light chain, or by separate expression of the two antibodies (each containing a different mutant H chain), followed by mixing of the purified antibodies under mild reducing conditions that preferentially reduce the disulfide bridges in the hinge region rather than the disulfide bond linking the CL and CH1 domains. Under carefully controlled conditions, properly assembled bsAbs can be produced at high yield, yet additional purification steps are usually needed to remove contaminating mispaired variants. Biparatopic hcAbs carry four antigen binding modules and thus are tetravalent, whereas conventional bsAbs carry only two antigen binding modules and thus are bivalent.

Biparatopic hcAbs have several inherent advantages over conventional bispecific antibodies (bsAbs) (Figure 8). Evolution has shaped a remarkably high stability and solubility of camelid VHH domains in the absence of a paired light chain (22, 24, 39). Our study shows that this unique biochemical property can be exploited to construct highly soluble, stable CD38-specific biparatopic hcAbs that induce potent CDC. Owing to their excellent solubility, nanobody-based biparatopic hcAbs are easier to construct, produce and purify at high yield than corresponding constructs based on conventional H + L chain antibodies. Biparatopic hcAbs are composed of two copies of a single polypeptide chain (Figure 8A). In contrast, conventional bsAbs are typically composed of two or more distinct polypeptide chains (Figures 8B,C) (40). The latter requires careful titration of two or more expression vectors and/or the use of dual cassette vectors in order to ensure expression in the appropriate molar ratios. In contrast, production of a biparatopic hcAb requires transfection of cells with only a single vector encoding a single heavy chain composed entirely of naturally highly soluble protein domains. A key structural advantage of a biparatopic heavy chain antibody over symmetric bsAbs (Figure 8B) lies in the high solubility of each VHH vs. the inherent instability of VH-VL pairing. For the proper assembly of bsAbs in the regular IgG format (Figure 8C), it is necessary to introduce mutations into the CH3 domains to promote pairing of two distinct H chains, resulting in asymmetric antibodies. Similarly, mutations need to be introduced into the CH1 and CL domains to promote the proper paring of H and L chains (41–43).

A potential advantage of biparatopic hcAbs and symmetric bsAbs over asymmetric bsAbs is their higher valency. Biparatopic hcAbs are tetravalent, i.e., they carry four antigen binding modules, each composed of a single highly soluble Ig-domain (Figure 8A). In contrast, bsAbs in the regular IgG format are bivalent, i.e., they carry only two antigen binding modules, each composed of two or more Ig-domains (Figure 8C). It is likely that oligomers are induced more effectively by tetravalent than by bivalent Abs.

Biparatopic hcAbs also have inherent advantages over HexaBody mutants. A mutated Fc domain carries a higher risk of inducing an antibody response than the parental WT IgG. Moreover, some HexaBody mutants show a tendency to spontaneously assemble into hexamers (19). Such spontaneous aggregation could result in enhanced uptake of these complexes by the reticuloendothelial system, thereby reducing their *in vivo* half-life. Therefore, HexaBody mutants E430G and E345K that do not induce any hexamerization in solution and whose hexamerization is fully dependent on target binding were selected for clinical use (19).

Our study has potential clinical relevance for multiple myeloma patients: The observation that circulating myeloma cells in patients that develop resistance to daratumumab express increased levels of complement inactivating cell surface proteins (CD55, CD59), suggests that CDC is an important tumor cytotoxic mechanism *in vivo* (44). Indeed, the finding that daratumumab displays higher CDC-inducing potency than other CD38-specific moAbs accelerated its path to clinical use (9, 18). Here, we demonstrate that the CDC-potency of daratumumab can be enhanced by complementation with a CD38-specific hcAb, provided that the latter recognizes a distinct, non-overlapping epitope of CD38. Future studies are needed to assess whether this enhancing effect by a CD38-specific hcAb also renders myeloma cells of patients that have become refractory to daratumumab susceptible to CDC. Moreover, we demonstrate that CD38-specific biparatopic hcAbs recognizing two distinct epitopes of CD38 display more potent CDC than daratumumab.

A potential limitation for biparatopic hcAbs with increased complement activation potential is the risk for killing CD38-expressing normal cells and for generating off-target cytotoxicity. CD38 is highly expressed by multiple myeloma plasma cells and a small subpopulation of regulatory T cells (Tregs) (45). CD38 is also found on natural killer (NK) cells, monocytes, B cells, and T cells of healthy donors (45). Treatment with daratumumab results in a preferential depletion of CD38+ immunosuppressive cells, with a concomitant increase in functional T-helper and cytotoxic T cells. It will be important to determine whether biparatopic hcAbs can mediate similar beneficial effects by preferentially killing CD38+ immunosuppressive cells.

In conclusion, our results underscore the advantages of using a heavy chain format with soluble nanobodies rather than pairs of VH and VL domains in antibody engineering. Moreover, our study highlights two new strategies for improving the benchmark antibody therapy of multiple myeloma: (1) complementing daratumumab with monospecific hcAbs, and (2) using biparatopic hcAbs as alternative therapeutics, e.g., in combination with other anti-myeloma drugs.

## Methods

### Cells

Human cell lines were obtained from the Leibniz-Institute DSMZ-German Collection of Microorganisms and Cell Cultures, Braunschweig, Germany (LP-1, ACC 41; CA-46, ACC 73). The CD38 gene was inactivated in LP-1 cells using CRISPR/Cas9 technology using a commercial double nickase plasmid (Santa Cruz sc-401117-NIC). CD38-negative cells were sorted on a FACS AriaII (Becton Dickinson).

### Construction of monospecific and biparatopic hcAbs

The coding region of selected nanobodies (WO 2017/081211) was subcloned using NcoI/PciI and NotI upstream of the coding region either for the hinge, CH2 and CH3 domains of human IgG1 (UniProt P01857) or hexahistidine and c-myc tags in pCSE2.5 vectors (46) (kindly provided by Thomas Schirrmann, Braunschweig). The amino acid sequence of the VHH-IgG1 junction is: VTVSSEPKTPKPQP-AAA-SDKTHTCPPCPAP where AAA is encoded by the NotI site. Biparatopic heavy chain antibodies were constructed by gene synthesis, fusing nanobodies WF211 and WF121 via a G4S_2_ linker, MU1067 and JK36 via a G4S_3_ linker and MU1068 and MU1067 via a G4S_7_ linker. Each nanobody dimer was flanked by NcoI and NotI and cloned as described above into the hIgG1 pCSE2.5 vector. Similarly, daratumumab scFv was generated by gene synthesis by fusing the VH domain and the VL domain (WO 2011/154453) via a G4S_3_ linker, flanked by NcoI and NotI sites and cloning into the hIgG1 pCSE2.5 vector.

### Construction of E345R HexaBody hcAbs

The E345R mutation was introduced into hcAbs by PCR-mediated mutagenesis. The mutation was verified by sequencing. In order to ensure that no other mutations were introduced into the vector, the human IgG Fc fragment encoding the E345R mutation was recloned into the pCSE2.5 vector using flanking restriction sites (NotI and XbaI).

### Production and purification of hcAbs

HcAbs were expressed in transiently transfected HEK-6E cells cultivated in serum-free medium (26, 47). Six days post transfection, supernatants were harvested and cleared by centrifugation. Recombinant proteins in cell supernatants were quantified by SDS-PAGE and Coomassie staining relative to marker proteins of known quantities: 10 μl samples of the supernatant were size fractionated side by side with standard proteins: m/M (amount loaded per lane in μg) bovine serum albumin (1/4), IgH (0.5/2), IgL (0.25/1), hen egg lysozyme (0.1/0.4). Yields of recombinant hcAbs typically ranged from 0.5–3 μg/10 μl. HcAbs were purified by affinity chromatography using protein G sepharose (GE healthcare).

### Complement-dependent cytotoxicity assays

Cells were incubated for 10–20 min at 4°C with hcAbs or moAbs before addition of human serum (10–15% v/v) and were then further incubated for 30–90 min at 37°C. Cells were washed and resuspended in PBS/0.2% BSA/propidium iodide before FACS analysis.

### C1Q binding assay

Cells were preincubated for 10–20 min at 4°C with hcAbs or moAbs before addition of human serum (10–15% v/v) and further incubation for 30 min at 4°C. Cells were washed and bound C1q was detected with FITC-conjugated rabbit anti-C1q (DAKO F0254) before FACS analysis.

## Author contributions

PB and FK-N conceived the project. FK-N wrote the manuscript. All authors established experimental procedures, performed experiments, reviewed, and approved the manuscript.

### Conflict of interest statement

CS is an employee of Ablynx NV, a Sanofi company, and owns shares and/or stock options with Ablynx/Sanofi. FH and FK-N receive a share of antibody sales via MediGate GmbH, a wholly owned subsidiary of the University Medical Center Hamburg-Eppendorf. PB, WF, LS, KS, SM, CS, and FK-N are co-inventors on a patent application on CD38-specific nanobodies. The remaining authors declare that the research was conducted in the absence of any commercial or financial relationships that could be construed as a potential conflict of interest.

## Abbreviations

Ab: antibody
bsAb: bispecific Ab
CDC: complement-dependent cytotoxicity
CDR: complementarity determining region
Fc: crystallizing fragment
hcAb: heavy chain antibody
Ig: immunoglobulin
kDa: kilodalton
NAD^+^: nicotinamide adenine dinucleotide
moAb: monoclonal antibody
Nb: nanobody
VH: variable domain of a conventional heavy chain
VHH: variable domain of a camelid heavy chain antibody
scFv: single chain variable fragment.
